# Impact of a Mediterranean-Inspired Diet on Cardiovascular Disease Risk Factors: A Randomized Clinical Trial

**DOI:** 10.3390/nu16152443

**Published:** 2024-07-26

**Authors:** Ana Rita Barbosa, Sandra Pais, Ana Marreiros, Marta Correia

**Affiliations:** 1 Comprehensive Health Research Centre—CHRC, Universidade de Évora, Colégio Luís António Verney, Gab:269-a, Rua Romão Ramalho 59, 7002-554 Évora, Portugal; 2 Algarve Biomedical Center Research Institute, Universidade do Algarve, FMCB Ed. Campus de Gambelas, 8005-139 Faro, Portugal; 3 Faculty of Medicine and Biomedical Sciences, Universidade do Algarve, FMCB Ed. Campus de Gambelas, 8005-139 Faro, Portugal; 4 CBQF—Centro de Biotecnologia e Química Fina—Laboratório Associado, Escola Superior de Biotecnologia, Universidade Católica Portuguesa, Rua Diogo Botelho 1327, 4169-005 Porto, Portugal

**Keywords:** mediterranean diet, cardiovascular risk factors, planetarian diet, visceral fat, waist circumference, randomized clinical trial

## Abstract

Cardiovascular diseases (CVDs) are the leading cause of death worldwide. This study focused on evaluating the impact of a Mediterranean-type diet combined with physical exercise on CVD risk factors of high-risk individuals. A randomized clinical trial (RCT) recruited individuals (≥50 years old) with no history of acute myocardial infarction, but with high CVD risk criteria according to the SCORE2/SCORE2 OP. Anthropometric and biochemical parameters were assessed at baseline and after 12 weeks of diet and exercise intervention. Participants were randomly assigned into 3 groups: no intervention group (Group 1a), physical exercise group (Group 1b), and physical exercise (±2 h/week) plus diet group (Group 2). Briefly, the dietary intervention was based on the principles of an isocaloric Mediterranean diet (MD), with seven main meals/week centered on plant-based foods (legumes and pulses). The combined effect of exercise and the diet showed significant decrease in WC (*p* = 0.002), BST (*p* < 0.001), visceral fat (*p* < 0.001), and TG (*p* = 0.029), compared with control groups. The intervention significantly increased legume intake (*p* < 0.001), as well as adherence to the MD, which associates with WC decrease (*p* = 0.024) and visceral fat (*p* = 0.017). A combined intervention of exercise and diet should be endorsed as an efficient modifier of cardiometabolic parameters.

## 1. Introduction

According to the World Health Organization (WHO), CVDs (cardiovascular diseases) are the number one cause of death worldwide [1] and, primarily coronary artery disease and stroke, continue to be recognized as a major contributor to disability and loss of years of life [2,3]. CVD cases worldwide have nearly doubled, from 271 million in 1990, to 523 million in 2019 [2]. In 2021, 9.44 million deaths were due to ischemic heart disease, and 3.87 million deaths to stroke, underscoring the urgent need for continued efforts in prevention and management of the modifiable CVD risk factors [4]. The underlying atherosclerotic process in acute myocardial infarction, the most severe of all manifestations of the disease, is typically initiated by the rupture of an atheromatous plaque, or erosion of the coronary artery endothelium, resulting in necrosis of cardiac cells [5]. This chronic and progressive inflammatory disease, associated with endothelial dysfunction, fosters the abnormal entry and deposition of LDL-C (low-density lipoprotein cholesterol) in the intima [6]. Hence, CVD risk factors include dyslipidemia (DL), diabetes mellitus (DM), and obesity, with a poor diet and sedentarism identified as one of the main contributors to this pathoetiological process [7].

The importance of overall dietary patterns, rather than individual nutrients or supplements, has become evident, strengthening the traditional Mediterranean diet (MD) as the dietary model that has gathered the largest body of evidence, as well as supporting lifestyle changes, in CVD prevention [8,9]. Indeed, it is well demonstrated that patients with an established CVD benefit greatly from adopting a healthy diet and sufficient physical activity as a first-line recommendation for clinical management, alongside lipid-lowering, blood pressure-lowering, and antithrombotic therapies [10,11,12]. Moreover, the recent CORonary Diet Intervention with Olive oil and cardiovascular PREVention (CORDIOPREV) trial showed that an MD was superior to a low-fat diet in secondary CVD prevention and resulted in 28% CVD event risk reduction [11]. Hence, robust evidence associating diet to atherosclerosis, and to CVD risk factors, is well established [13,14,15,16,17,18,19,20,21,22,23,24,25]. The most important dietary risks include a low intake in whole grains, fruits, fiber, legumes, nuts and seeds, omega-3 fatty acids, polyunsaturated fatty acids, vegetables, milk, and calcium, and a high intake of sodium, trans fats, red or processed meat, and sugar-sweetened beverages [26].

Additionally, programs promoting the MD, with or without physical activity, or other joined interventions, reduce all-cause mortality and non-fatal myocardial infarction in patients with increased cardiovascular risk [14]. Indeed, it has been demonstrated that one standard deviation increase in the most frequently used MD score is associated with a 29% relative reduction in CVD risk, which is in line with what is demonstrated in two RCT [8]. Likewise, the DASH diet (dietary approaches to stop hypertension) and the fruit and vegetable-enriched diet were also able to reduce the risk of the disease by 10.3% and 9.9%, respectively [23], most probably due to the improved endothelial function, and the decreased serum levels of oxLDL and LDL-c [27,28]. Interestingly, the MD has undergone a progressive evolution over the past 60 years, and it is, nowadays, of even higher importance, since it is evolving from a healthy dietary pattern to a recognized sustainable dietary pattern [29]. Additionally, an energy-reduced MD has been suggested as an effective strategy for weight loss and CVD prevention; results from a PREDIMED trial, which did not introduce any caloric reduction in the MD, support an energy reduction as a means to CVD prevention [30]. Moreover, amongst plant-based diets, current evidence suggests that the Mediterranean and vegetarian diets are associated with numerous health benefits, including a lower risk of CVDs, which seems to be related with their high content of dietary fiber, complex carbohydrates, vitamins, minerals, polyunsaturated fatty acids, and phytochemicals [31]. Indeed, Le Ma and coworkers [32] showed that a higher intake of isoflavones and tofu was associated with a moderately lower risk of developing coronary heart disease, and in women, the favorable associations of tofu were more pronounced in young women or postmenopausal women without hormone use, which opens new possibilities of the use of bioactive foods integrated into healthy plant-based diets, such as the MD, adding nutritional support in the prevention of CVDs [31].

Regarding physical exercise, there are no long-term RCTs on the effect of physical activity interventions on CRFs, although observational studies show that higher physical activity levels are associated with lower risk of CVDs [33,34]. A recent systematic review showed that initiating an active lifestyle was associated with a 45% relative risk reduction compared with a persistently inactive lifestyle, supporting that combined interventions [35] including Mediterranean-type diet together with physical activity impact body fat mass, visceral mass, and lipid profile, and should be considered as an effective way to modify CRFs in high-risk patients. Indeed, very recently, it was shown that in patients with established CVDs, a combined Mediterranean diet and physical activity intervention is cost saving and highly cost-effective compared with usual care, reinforcing the importance of combined strategies [36]. These findings strongly advocate the incorporation of lifestyle interventions as integral components of care for all patients with CVDs.

To our knowledge, an RCT using an MD, with no energy restriction, enriched with plant-based food items such as legumes, increasing the number of vegetarian meals combined with planned and supervised physical exercise, has not yet been conducted. Moreover, evidence is still scarce regarding these combined interventions on CRFs, justifying a multicomponent strategy. Hence, this study investigated the effects of a multicomponent program conveying an MD-type intervention with education sessions and exercise program in modifying high-CVD-risk body composition, blood biochemical analysis, and eating behaviors. Moreover, such an RCT, evaluating a more vegetarian version of the MD, combined with physical exercise in high-risk individuals, is crucial for providing comprehensive, evidence-based strategies to prevent CVDs, aligned with modern health and environmental priorities. Additionally, older populations, more at risk of CVDs, are more likely to experience cardiovascular events over a shorter period, which allows us to observe the effects of interventions more clearly and within a feasible study duration. Also, older people have often been excluded from clinical trials due to age, multimorbidity, and disabilities, and the external validity of many trials may be questioned, as individuals participating in trials are generally less complex than many patients seen in geriatric clinics [37]. Moreover, it is expected that older adults, with the same risk of CVDs (high risk) might have more homogenous baseline health conditions related to their risk, reducing variability and allowing for clearer interpretation of results.

## 2. Materials and Methods

The study was conducted according to the 2010 Consolidated Standards of Reporting Trials (CONSORT). The study protocol presented was approved by the Ethics Committee of the Nova Medical School, Faculty of Medical Sciences, Nova University of Lisbon (Nº. 22/2023/CEFCM). The trial was conducted following the Declaration of Helsinki and was registered at the ClinicalTrials.gov ID: NCT06113484.

### 2.1. Study Design and Eligibility

The intervention was conducted between July and December 2023 in the Algarve region. Adults aged 50 years or older, with high risk level in the Systematic Coronary Risk Evaluation (SCORE2) or the Systematic Coronary Risk Evaluation Older Person (SCORE2 O.P.) cardiovascular risk algorithm, were recruited from the community via their primary healthcare centers, flyers, or dissemination materials given out in local senior universities and public health communities. The exclusion criteria referred to individuals with a history of acute myocardial infarction, with cognitive impairment (Montreal Cognitive Assessment (MoCA)), with type 1 DM or insulin dependence, with a pacemaker device, currently performing formal exercise sessions for more than 30 min per week, classified as class III or IV angina according to the Canadian Cardiovascular Society criteria, classified as showing class III or IV symptoms according to the New York Heart Association criteria, with type 2 myocardial infarction, with uncontrolled/symptomatic cardiac arrythmia with hemodynamic impact, with severe and symptomatic aortic valve stenosis, with uncontrolled and symptomatic heart failure, with active myocarditis, pericarditis, or endocarditis, with acute aortic syndrome, with known or suspected desiccant aneurism, with acute systemic infection, who were receiving nutritional supplementation, following a vegetarian or strict vegetarian dietary pattern, or on a nutritionist counselor.

### 2.2. Randomization and Study Groups

**Figure 1:**
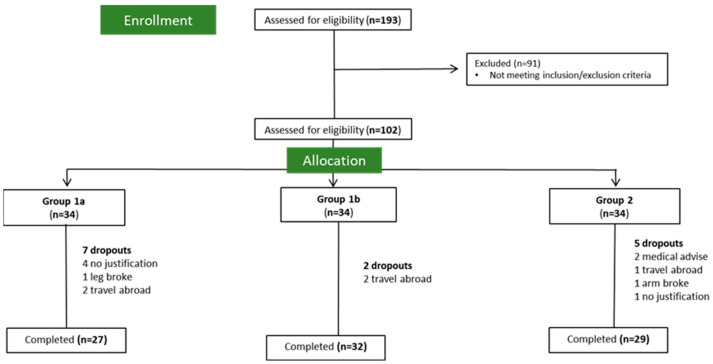
Flow chart of participant recruitment and allocation.

The study was a non-blinded RCT with a 12-week combined intervention of an MD-type diet and a controlled and planned physical exercise, with 3 parallel arms and a 1:1:1 allocation. Eligible participants were randomized using computer-generated numbers. Therefore, the study encompassed: (a) a negative control (Group 1a), with no intervention; (b) a positive control (Group 1b) with physical exercise intervention, supervised in loco with exercise specialists (2 x/week, 2 h), and no dietary intervention; and (c) an intervention group (Group 2), conveying a diet plus the same physical exercise plan. In Figure 1, we present the flow chart of participant recruitment and allocation. This intervention study aimed to evaluate the clinical benefit in adding a specific diet (an MD-type diet) to a physical exercise plan. To that end, the proposed study evaluated the effect of the physical exercise isolated from the diet to: (1) exclude the putative beneficial potential of the physical exercise of the combined program, used to evaluate the impact of exercise on CVD risk factors; and (2) better evaluate the combined effect of the diet and physical exercise.

### 2.3. Intervention Strategy

The nutritional intervention was designed based on the principles of the Mediterranean dietary pattern and the planetary diet (the EAT-Lancet Diet) [38,39]. The Group 2 participants were instructed to make significant changes to their diets as follows: (a) to include more vegetables, fruits, seeds, and, in general, less processed meals (≤1, every 2-weeks); (b) the number of meals were adjusted to three to four meals per day, depending on the existing scheme of meals; (c) regarding the main meal (lunch and dinner), legumes and pulses, such as chickpeas, all types of beans, lentils, and broad beans, were to be prioritized as the source of protein every dinner, whilst at lunch, fish, lean meat (chicken, poultry), or eggs, ought to be included. Therefore, the dietary intervention conveyed 7 vegetarian meals per week (dinner); (d) participants were also instructed to exclude the consumption of red and/or processed meats during the 12-week intervention period, as well as to respect an overnight fasting of at least 12 h, and a maximum of 16 h, leaving enough time to organize 3 to 4 meals a day; (e) the inclusion of whole grains (whole pasta, whole rice, whole bread) instead of refined grains; (f) increasing vegetable intake (at least 5 portions/day); (g) one low-fat dairy source per day (maximum); and (h) limit potato consumption to twice a week. Subjects were not instructed to count calories, and personalized meal plans were not generated. Recommendations reflect the EAT-lancet diet and Mediterranean diet features. The study produced and divulged different support materials, which were provided to each participant, such as book recipes, a document with recommendations for food sustainability, and, finally, a summary of all recommendations provided in the consultation (see complementary files). Additionally, the study included a two-hour group culinary workshop for Group 2, and to optimize adherence to the nutritional program, participants had monthly in-person nutrition consultations and biweekly follow-ups via telephone. In these appointments (in-person or virtual), the nutritionist aimed to better understand the possible difficulties experienced by the participants, as well as provide suggestions for improvement. The control groups, 1a and 1b, were also given an educational food and nutrition group session—recommendations of the Mediterranean Diet Wheel [40,41]. All three groups had an educational session conducted by a clinical psychologist specialized in behavioral modification on the importance of lifestyle changes, focused particularly on physical exercise and diet, to prompt the needed lifestyle changes.

### 2.4. Studied Outcomes

A team of trained nutritionists and clinical analysis technicians accessed primary (weight, BMI, and waist circumference) and secondary (analytical values, other body composition, anthropometric measurements, and adhesion to the MD) outcomes at baseline and week 12 after the programmed intervention.

#### 2.4.1. Anthropometric Parameters and Body Composition Assessment

The evaluation of body weight (kg), BMI (kg/m^2^), percentage of fat mass, muscle mass (kg), and visceral tissue was performed using the Medical Body Composition Analyzer—SECA^®^ mBCA 514/515 equipment (Seca hmbh & co.kg, Hamburg, Germany). The waist circumference (WC) and the arm circumference (AM) were measured using a flexible anthropometric measuring tape from CESCORF (Porto Alegre, Brazil); the bicipital (BSF) and tricipital skinfolds (TSF) were measured with the INNOVARE 3 skinfold caliper from CESCORF. It was used in the methodology established by the International Society for the Advancement of Kinanthropometry [42].

#### 2.4.2. Dietary Assessment

To evaluate adherence to the MD, the PREDIMED tool was used. Furthermore, to assess adherence to the proposed diet before and after the intervention of this study, two additional questions were posed to all participants: (1) How often do you consume a vegetarian meal per week with legumes as a protein source?; and (2) Do you practice a daily overnight fast of at least 12 h? With these two questions, we assessed if the study objectives were “achieved”. Answers to question (1) were considered positive and scored 1 when the number of vegetarian meals increased from baseline to post intervention and scored 0 when they decreased. Answers to question (2) were scored and were considered positive and scored 1 when the number of overnight fasting hours increased from baseline to post intervention and scored 0 when they did not. To achieve a positive result for the purposes of the study objective, participants had to answer affirmatively to both questions, 1 point (yes); otherwise, they were scored 0 points (no). Additionally, we also assessed putative dietary changes inspired on the EAT-Lancet diet and Mediterranean recommendations addressing the following questions: (3) Do you consume at most one low-fat dairy product/day?; (4) Do you consume 30 grams of nuts/day?; (5) Do you consume whole grains everyday?; (6) Do you consume vegetables in both main meals?; (7) Have you excluded red meat from your diet?; (8) Do you consume alcoholic beverages in moderation?,; (9) Do you consume potatoes at most twice a week?; and (10) Do you have four meals/day?. The answers scored 1 when considered positive and scored 0 when if negative.

#### 2.4.3. Analytical and Biochemical Assessment

The COBAS b 101 system (ROCHE diagnostics, Rotkreuz, Switzerland) was used for the biochemical evaluation (lipid profile, C-reactive protein (CRP), and HbA1c) of all participants after a minimum of 12 h overnight fast.

#### 2.4.4. Evaluation of Cardiovascular Disease Risk

A trained cardiologist assessed all participants for baseline cardiovascular risk. The SCORE2 or SCORE2 O.P. algorithm was utilized [43,44].

### 2.5. Power Analysis and Sample Size

The G*Power software version 3.1.9.7 was used to calculate the sample size, taking into account the following variables of interest: body weight, waist circumference, and body fat percentage (12). A minimum statistical power of 80% was used, assuming an average effect size (between 0.25–0.30) and a significance level of 0.05. In order to detect differences in the variables of interest, the minimum sample size required was 24 individuals per group, for repeated measures at two points in time within each factor. Considering a maximum expected loss of between 15% and 20% over the course of the study, the final sample size was set at between 84 and 90 individuals, evenly distributed between the three control and intervention groups, which on average would give around 28 individuals per factor. The final sample size was 88 individuals with the following distribution: 27 for Group 1a, 32 for Group 1b, and 29 for Group 2.

### 2.6. Statistical Analysis

The data collected were statistically analyzed using IBM SPSS version 29 software. Descriptive analyses were carried out using mean and standard deviation, median, and interquartile range for quantitative variables. Frequencies were used for categorical variables. Descriptive analyses were carried out using mean and standard deviation, median, and interquartile range for quantitative variables. Frequencies were used for categorical variables. Using the logic of paired samples (before and after the intervention), and based on the initially pre-defined group category, a bivariate analysis was carried out. The normality of the data was first tested using the Kolmogorov–Smirnov test or the Shapiro–Wilk test. This procedure defined whether a parametric or non-parametric approach was used. The comparison of variables in the same group (baseline vs. 12 weeks) was carried out using parametric tests (Student’s *t*-test for paired samples) and non-parametric tests (Wilcoxon Signed-Rank test). Spearman’s non-parametric coefficient was used to test the correlation between quantitative variables. For all inferential processes, *p* < 0.05 was considered statistically significant for results. For comparisons between the study groups (Group 1a; Group 1b; and Group 2), parametric ANOVA tests were used with post-hoc tests if *p* < 0.05, and non-parametric Kruskal–Wallis tests as appropriate, considering the assumptions of normality. In this context, the initial variables were transformed into a rate of change:

$$∆=\left[\left(Post\;Intervention-Pre\;Intervention\right)/Post\;Intervention\right]\times 100$$

If ∆ < 0, post intervention decreases, if ∆ > 0, post intervention increases, and if ∆ = 0, post intervention remains the same. This transformation solves the problem of heterogeneity at the time of the initial intervention and considers that the starting points of pre intervention values of the individuals are different, as expected.

## 3. Results

### 3.1. Baseline Sample Characterization

The study included a total of 102 adults, with a mean age of 70.1 ± 7.9 y, diagnosed with high cardiovascular risk that followed a randomization into one of three groups: Group 1a (*n* = 34), Group 1b (*n* = 34), and Group 2 (*n* = 34). The clinical and nutritional characterization of the initial sample, including the 14 participants that later dropped out, is described in Table S1, in the Supplementary Materials.

Out of the 88 participants in the study (102 participants minus the 14 that dropped out), the majority, 73.9%, were women, 63.6% were retired, 63.6% were married, and 62.5% were living with their partner. In terms of educational attainment, 31.8% of participants completed primary education, 15.9% completed secondary education, 13.6% completed tertiary education, and only 11.4% completed higher education. All socioeconomic characteristics of all study participants are detailed in Table S2.

Regarding clinical history, most participants had diagnoses of dyslipidaemia (69.3%) and hypertension (68.2%), with a higher percentage of hypertensive individuals allocated to Group 1b (81.3%), resulting in a higher intake of antihypertensive medication (81.3%), and dyslipidaemias allocated to Group 2 (79.3%). The use of statins was similar in both groups. Blood pressure values also showed similar means across the three groups and were assessed (Table S3). Several participants had a family history of myocardial infarction or sudden death of a parent or other first-degree relative before the age of 55/65: nine participants in intervention Group 2 (31.0%); eleven participants in Group 1b (34.4%); and nine participants in Group 1a (33.3%). The prevalence of cardiac insufficiency, arrhythmia, and stroke was assessed; however, their prevalence was low in the study sample. Although smoking habits were not prevalent in any of the three groups, 50% of the participants in Group 1b reported alcohol consumption. Regarding another evaluated CRF, participants in intervention Group 2 (20.7%) were less sedentary compared to the others.

When evaluating BMI (*p* = 0.711) at the beginning of the study (Table S4), participants were mostly classified as overweight (46.6%) and obese (31.8%). The mean body weight was 71.3 kg ± 13.9 and did not show significant differences between groups (*p* = 0.599). High percentages of body fat (*p* = 0.849) were observed in all three groups (over 40%), while the amount of muscle mass (*p* = 0.294) did not exceed 26%. Regarding visceral fat, Group 1b had a higher average than the other groups, but also without significant differences. Most men had WC values below the cardiovascular risk threshold (<102 cm) of 65.2%, while most women had significantly elevated values (>88 cm) of 66.2%. Finally, when measuring skinfold thickness (bicipital (BSF) and tricipital (TSF)), participants in the intervention group had thicker skinfolds compared to the other groups (BSF: *p* = 0.754; TSF: *p* = 0.336).

At baseline, there were no statistical differences between the groups regarding serum total cholesterol (*p* = 0.398), LDL-c (*p* = 0.460), HDL-C (*p* = 0.649), TG (*p* = 0.978), HbA1c (*p* = 0.732), and CRP (*p* = 0.131). At the beginning of the study, participants, on average, had elevated total serum cholesterol (192.6 ± 43.6 mg/dL). However, when distributed into groups, most participants in Group 1a (63.0%) had a total cholesterol below 190 mg/dL, while in Group 2, 65.5% of participants had higher total cholesterol. Group 1b had an even distribution (50.0% vs. 50.0%). At baseline, the mean LDL-c was 108.1 ± 38.8 mg/dL, and statistically, no significant differences in LDL-c were observed between the three groups. However, when analyzing the group means, Group 2 presented a higher average than the other groups. Importantly, in all the three groups, more than 50% of participants had values below the recommended upper limit. Both men and women had HDL-c values within the reference range (♀ > 45 mg/dL; ♂ < 40 mg/dL), as observed in Table S5. Although not statistically significant, participants in Group 1a showed higher TG values, followed by Group 2, and, lastly, Group 1b. However, when evaluated according to the reference range, most participants in both groups had values below the recommended limit. At baseline, 13.6% of participants had HbA1c values above 6.5%, compared to 50.0% with values equal to, or less than 5.6%. Finally, the CRP in more than 70% of participants did not present any sign of acute inflammation, harboring CRP levels below <0.03 mg/L (*p* = 0.131). The adherence to DM (Table S6) showed that most participants exhibited moderate to high adherence (participants were recruited from the Algarve region, where the MD is well engrained in their gastronomy culture), with no significant differences between the groups at the baseline (*p* = 0.552).

### 3.2. Effect of the Intervention on the Body Composition and Anthropometry Assessment

In the evaluation of body weight, body mass index, percentage of body fat, and percentage of muscle mass, all three groups showed statistically significant differences over the course of the study. However, when assessing differences between the groups at the end of the study, these were not observed, as described in Table 1. When evaluating WC, no statistically significant differences were found between the groups. However, the intervention group, Group 2, was the only group that showed a significant decrease (95% CI: −0.065, −0.020; rate of change = −2.12%) compared to baseline. The same tendency was observed in the variable BSF (95% CI: −1.450, −0.400; rate of change = −19.38%). Regarding the assessment of visceral fat, in Group 1a, we observed that the rate of change had a significant increase at the end of the study. On the other hand, both Group 1b (95% CI: −0.400, 0.000; rate of change = −35.19%) and Group 2 (95% CI: −0.650, −0.250; rate of change = −31.46%) showed a decrease in the mean value, with only Group 2 achieving a statistically significant result. At the end of the study, statistically significant differences were observed between the study groups for TSF, due to the comparison between Group 1b with Group 2, with the latter group exhibiting a decrease with a higher rate of change. When adjusting the variables for the “statins” factor, no differences were observed, meaning that taking statins did not influence the observed results.

### 3.3. Effect of the Intervention on the Biochemical Parameters

After the intervention, the mean values of total cholesterol decreased by 11.53 mg/dL in Group 1b (95% CI: 1.274, 21.788; *p* = 0.029) and 6.41 mg/dL in Group 2 (95% CI: −7.587, 20.414; *p* = 0.356). On the other hand, the mean cholesterol in Group 1a increased by 1.81 mg/dL (95% CI: −12.641, 9.012; *p* = 0.733). However, no statistically significant differences were observed between the three groups (*p* = 0.312). Regarding LDL-c values, Group 1a showed a growing trend in the median value (95% CI: 1500, 15,500; rate of change = 6.14%), but without statistical power. In contrast, the exercise group (95% CI: −13,500, 2500; rate of change = −10.47%) and the exercise and diet group, Group 2 (95% CI: −18,500, 0.500; rate of change = −9.48%), showed a decrease in LDL-c values compared to the beginning of the intervention, also with statistical significance in Group 1b. There were statistically significant differences between the study groups (*p* = 0.010) and Group 1a with Group 2, and comparing Group 1b with Group 1a (Table S7).

HDL-c values were increased only in the intervention group (95% CI: −3.647, 0.888; rate of change = 2.06%); however, this was without statistical significance. Surprisingly, significant negative results were observed in Group 1b (95% CI: 0.952, 5.673; rate of change = −6.17%), meaning that HDL-c decreased after the exercise intervention. In that sense, we checked if differences between genders could explain these results, but no significant differences were found (*p* = 0.369). Additionally, participants with higher HDL-c were associated with lower values of body weight (r_s_ = −0.326; *p* = 0.002), BMI (r_s_ = −0.222; *p* = 0.038), WC (r_s_ = −0.351; *p* < 0.001), AM (r_s_ = −0.323; *p* = 0.002), muscle mass (r_s_ = −0.317; *p* = 0.003), and visceral fat (r_s_ = −0.352; *p* < 0.001).

The intervention with diet and exercise also proved effective in significantly decreasing TG values (95% CI: −35.000, −1.000; rate of change = −24.36%). On the other hand, participants of Group 1b showed an increase in the median (95% CI: −12.000, 23.000; rate of change = 4.64%), but without significance. Elevated TG values were positively associated with body weight (r_s_ = 0.258; *p* = 0.015), BMI (r_s_ = 0.355; *p* < 0.001), WC (r_s_ = 0.348; *p* < 0.001), arm circumference (r_s_ = 0.227; *p* = 0.034), BSF (r_s_ = 0.220; *p* = 0.041), fat mass (r_s_ = 0.384; *p* < 0.001), and visceral fat (r_s_ = 0.271; *p* = 0.011). Interestingly, they were negatively associated with legume/pulses consumption (r_s_ = −0.248; *p* = 0.020).

Significant differences were observed between the groups in the HbA1c variable (*p* = 0.047). This variable was positively associated with all anthropometric variables except body mass (r_s_ = −0.215; *p* = 0.044), as observed in Table S7. These differences were reflected by the comparison of Group 1a with Group 2.

Finally, no statistically significant differences were observed in the CRP variable, nor any association with other variables. The results are shown in Table 2 and Table S7, respectively.

When adjusting the variables for the “statins” factor, no differences were observed, meaning that taking statins did not influence the observed results.

### 3.4. Effect of the Intervention on the Adherence to the Proposed Diet

All participants in the intervention group demonstrated improvements in their diet; however, only 41.4% were able to adhere to all the dietary goals proposed at the beginning of the intervention. Following-up the evaluation of the participants a significant association between muscle mass gain (*p* = 0.002) and TSF (*p* = 0.032), was found, as described in Table S8. When evaluating possible associations (Table S7), these participants tended to have lower values of body weight (r_s_ = −0.222; *p* = 0.038), BMI (r_s_ = −0.229; *p* = 0.032), WC (r_s_ = −0.315; *p* = 0.003), arm circumference (r_s_ = −0.302; *p* = 0.004), and visceral fat (r_s_ = −0.282; *p* = 0.008).

Regarding the consumption of legumes and pulses (Table 3) as an important plant protein source, only Group 2 showed a significant increase (*p* < 0.001). Participants with higher legume consumption showed lower values of WC (r_s_ = −0.241; *p* = 0.024), visceral fat (r_s_ = −0.253; *p* = 0.017), and TG (r_s_ = −0.248; *p* = 0.020), as observed in Table S7. With respect to compliance with the suggested fasting period of at least 12 h, 11 participants (XX%) started this practice, increasing their overnight fasting period, whilst 4 (XX%) discontinued it from their daily routine. The practice of overnight fasting (12 h) was not significantly associated with any of the evaluated outcomes (Table S7).

When assessing adherence to the MD with PREDIMED score (Table 3), no significant differences were observed between groups (*p* = 0.061). However, in Group 2, the intervention led to a significant increase to MD adherence (95% CI: 0.000, 1.500; rate of change = 7.64%). On the other hand, a decrease was observed in Group 1b (95% CI: −1.000, 0.500; rate of change = −4.99%), but without statistical significance. Participants showing higher adherence to the MD according to PREDIMED had (Table S7) lower values of BMI (r_s_ = −0.268; *p* = 0.012), WC (r_s_ = −0.279; *p* = 0.008), AM (r_s_ = −0.288; *p* = 0.006), BSF (r_s_ = −0.389; *p* < 0.001), percentage of body fat (r_s_= −0.318; *p* = 0.003), and also HbA1c (r_s_ = −0.255; *p* = 0.017). Because PREDIMED might not be sensitive enough to identify dietary changes, we questioned participants before and after the intervention and, interestingly, significant increases in nuts, legumes, whole grains, and vegetables, as well as significant decreases in red meat intake, were observed (Table 4).

## 4. Discussion

This study sought to address the relation between an MD, a plant-based diet, and regular practice of physical exercise, in the primary prevention of CVDs in high-risk individuals. The intervention was centered on a broader interpretation of the MD but encompassing exercise and dietary changes expected to resonate with the Portuguese tradition and gastronomy. The dietary and nutritional recommendations endorsed in the study stemmed from the principles of the MD and the EAT-Lancet diet [38,40,41]. One of the main objectives of our intervention was to decrease the consumption of animal items, such as meat and processed meats, decreasing the total fat provided by animal sources. Considering the age of the study sample (70.1 ± 7.9 years) and the low literacy in health, promoting changes in the eating behaviors was often difficult to attain, and some participants struggled to fully embrace all the dietary suggestions. Nevertheless, all participants showed improvements, particularly regarding the consumption frequency of legumes (as protein sources), an increase in vegetables and whole grain cereals, and replacing one main meal of animal protein with a vegetarian one. In this context, 41.4% participants registered full changes in their diet, substituting/introducing a traditional dinner/night snack for a vegetarian dinner every day of the week throughout the study; noticeably, almost 70% increased their vegetarian meals, but did not achieve the recommendation of a vegetarian meal per day. Dietary changes, although difficult to implement, are possible, and some studies have demonstrated their feasibility, reporting changes towards a more vegetarian pattern—a green-MD—in similar population groups [45]. Interestingly, and although no significant differences were found between the groups, the study shown an increase in adherence to the MD in the intervention group, shifting from moderate to high adherence. This might be related with increased intake of pulses, fruits, and vegetables, and the decreased intake of red meat, which was part of the nutritional recommendation. Indeed, significant increases in nuts, legumes, whole grains, and vegetables, as well as significant decreases in red meat intake were observed, alongside a decrease to four meals a day. Altogether, these dietary changes might have contributed to the clinical and analytical results. A significantly higher intake of legumes, naturally rich in fiber and isoflavones, has been shown to decrease the risk of CVDs. Indeed, isoflavone intake (tofu, but not soy milk) was demonstrated to be inversely associated with CHD with one ≥1 serving/week [32], encompassing around 100 mg of isoflavones per portion, which is more more than pulses, although the latter are still an important source [46]. This association seems to be of particular significance in postmenopausal women without hormone use (*P*_interaction_ = 0.002), which the women recruited in this study were. Interestingly, participants of Group 2, who have reported a significantly higher intake of legumes/pulses compared with both control groups, were associated with a significant increase in muscle mass and a significant decrease in TSF (fat). Also, these participants presented lower values of body weight, BMI, WC, arm circumference, and visceral fat mass. Hence, participants with higher legume consumption tend to present lower WC, visceral fat, and triglycerides, which is suggestive of the importance of a more plant-based diet centered around the MD for a healthier metabolic profile in high-risk CVD patients [47].

In addition to diet, this study has recommended an increase of the length of overnight fasting (from the 6–7 h reported by the participants) to at least 12 h, as it has been demonstrated that an overnight fasting period is associated with a better glycemia and metabolic control [48].

This study did not find significant benefits of a 12 h overnight fasting period in modifying CRFs. This might be explained due to participants’ inaccurate reporting, or because 12 h fasting may not be sufficient to deplete all hepatic glycogen, and consequently, the remaining positive metabolic processes are not observed in this population. In addition to the significant improvements of some anthropometric parameters (reduction of body weight, BMI, AM, WC, skinfold thickness (BSF and TSF), fat mass, and visceral fat, and an increase in muscle mass), as previously described, Group 2 presented important analytical changes, with a significant decrease in serum triglycerides. Indeed, a recent review and meta-analysis demonstrated that the adherence to an MD resulted in an improvement in various risk factors for metabolic syndrome, as well as significantly lowering the incidence of CVDs [49]. In the intervention group (Group 2), no statistical significance was observed in the values of total cholesterol and LDL-c; however, when the variation rates were analyzed, decreases of −4.56% in the total cholesterol and −9.84% in the LDL-c values were observed, suggesting that the nutritional intervention had a positive impact on these variables, despite not reaching statistical significance. It is noteworthy that statins, which were taken by more than half of participants (51.1%), did not interfere with the LDL-c levels presented by participants, meaning that the differences between groups cannot be explained by statins. The significant decrease in triglycerides that occurred in the intervention group (Group 2) highlights the importance of the combined intervention (diet + exercise) in modifying serum lipid profile. Atherogenic dyslipidemia, which is a common lipid disorder associated with increased risk of CVDs, is characterized by high concentrations of serum triglycerides, low concentrations of HDL-c, and normal values of plasma LDL-c, stressing the importance of this parameter in the etiopathology of CVDs [50]. In this study, the significant decrease in triglycerides (almost 25%), coupled with the observed trend in the increase in HDL, reinforces the importance of a combined diet and physical exercise in CVDs. The decrease in triglycerides is already described in other RCTs using a standard MD, but not to the extent we have shown in this study [51].

The metabolic changes induced by both diet and physical exercise might also be due to adipokine modulation, such as adiponectin, leptin, and visfatin levels, and it would have been further elucidating to have shown these biomarkers for CVDs in older populations [52]. Nonetheless, there expected to be a greater variability in adipokine levels amongst older individuals, as they are dependent on comorbidities and medication, which, alongside the lack of standard cut-off levels for this specific population, warrants further large-scale prospective studies to validate their predictive value [53].

As mentioned, Group 2, was the only group showing a significant reduction in the WC and BSF. Visceral fat was also a variable that showed a significant decrease in this group (significance was also observed in Group 1a); however, since only Group 2 showed a significant decrease in WC, one of the parameters associated with cardiovascular risk, it is interesting to note that this was also the group that exhibited the greatest reduction in visceral fat (a loss of around 30%). A previous RCT, assessing the impact of an isocaloric MD and a plant-based polyphenol-enriched MD, with reduced red meat consumption (green-MED), showed results in line with ours [45,49]. Although both diets have led to a moderate decrease in body weight and WC, the green-MED resulted in a much more significant decrease, doubling the loss of visceral adipose mass compared with the non-green-MD [45]. These results support our dietary strategy, a more plant-based MD, to achieve lower CRFs.

Surprisingly, the control groups (Group 1a and Group 1b) showed a decrease in body weight, BMI, body fat, and TSF, and one cannot rule out the influence of the given dietary educational sessions on these variables. Another possible explanation might be that seasonal changes and lifestyle behaviors that came along with summer (study included summer months) impacted eating and exercise behaviors. As mentioned, this study was conducted in the Algarve region, specifically in the cities of Quarteira, Albufeira, and Faro, where fish intake increase is associated with the season [54,55]. Also, the climate of the Algarve allows the population to enjoy a wide variety of vegetables and fruits that only thrive in warm climates [56]. In Group 1b, we can further highlight the positive impact of physical exercise on the improvement of the aforementioned parameters, which is consistent with scientific evidence [57,58,59]. However, the decrease in HDL-c concentration in this group is somewhat puzzling and is not aligned with the literature [59]. Indeed, physical exercise is strongly recommended as a first-line treatment for elevated cholesterol levels [60], and as an effective booster, specifically, of HDL-c levels ([61,62]). Since Group 1b includes premenopausal women, we attempted to understand if there was a gender association responsible for the variation in HDL-c, due to the sudden lack of hormonal protection, and surprisingly, women dropped double the HDL-c variation levels compared to men, although without reaching statistical significance. It is worth noting that there is no single kind of HDL cholesterol particle, and some types are spherical while others are doughnut-shaped, [63] which will interfere with LDL protection and impact inflammation in artery walls. Another possible explanation could be that Group 1b registered significant losses in total cholesterol, which might account for a virtual loss of HDL-c. Nevertheless, it is interesting to note that there is a gender difference, which might justify increasing the sample size to more accurately assess.

Although weight loss and subcutaneous fat loss were registered, no significant positive changes were observed in the control groups regarding WC and serum triglycerides, which are intimately associated with primary prevention of cardiovascular disease. This prompts us to infer that both weight and subcutaneous fat loss are not as specific as triglycerides and visceral mass, in CRF assessment. Furthermore, when evaluating the biochemical profile of participants with weight loss (Group 1a and Group 1b), it was shown that, despite improvements in some anthropometric parameters, it did not translate to a better lipid profile, except for total cholesterol in the exercise group.

In this study, the results obtained with diet and a physical exercise plan led to similar outcomes, except for waist circumference, visceral fat, and serum triglycerides, which presented significantly better results in Group 2, demonstrating the potential effect of the combined diet with exercise. It is noteworthy that several nutraceuticals and bioactive molecules (vitamin D, isoflavones, flavanols) present in food items traditionally found in the MD, such as oregano and legumes, or not so common in the MD, like curcumin, soy products, and tea, have shown significant anti-inflammatory, anti-oxidant, and bioactive properties in CVD prevention, thus showing a potential use of the MD along with these particular items [64]. It is also important to acknowledge that the MD has been shown to modulate the gut microbiota and to be associated with gut microbiota that decreases gut permeability, modulates inflammation, and increases variability [65]. In this study, we did not have the possibility to conduct a gut microbiota analysis, but it could have shed some light on the mechanism by which our proposed MD, plus a physical exercise integrative program, successfully decreased CVD risk factors.

This study has limitations, such as its small sample size and short intervention time, that might have limited some inferences. Moreover, more than half of the participants were medicated with statins, which directly interferes with lipid metabolism. Nevertheless, statistical analysis excluded any influence of statins on clinical results. It is also worth noting that a dietary intervention group, without exercise, could have added some additional insight, although the intention of this study was to assess the combined effect of exercise and diet. As positive aspects, we highlight the fact that this intervention was conducted in a population with a high risk of CVDs, a group that needs urgent preventive strategies for better cardiovascular outcomes. Furthermore, the study being conducted in different centers allowed for greater heterogeneity in study.

## 5. Conclusions

The present study demonstrated that a Mediterranean-type diet was effective in reducing cardiovascular risk factors, such as WC, visceral fat, and plasma triglyceride levels. These results are suggestive that a diet inspired by the MD, enriched in vegetarian meals, with decreased meat consumption, excluding red meats, reducing starchy foods, and promoting the consumption of foods rich in polyunsaturated fatty acids, whole grains, fruits, and vegetables, impacts the cardiometabolic health of individuals at high risk of CVDs. Furthermore, physical activity, by itself, was not able to demonstrate the positive metabolic and anthropometric changes observed in Group 2, which clearly emphasizes the importance of a multicomponent strategy to address a complex and multivariable pathology. Additionally, this RCT stresses the importance of an assessment of body composition that values other parameters, beyond body weight, BMI, and subcutaneous fat, since the latter did not translate into high precision variables in detecting cardiovascular risk. Findings from this RCT might influence clinical practices and public health policies, ultimately contributing to reduced cardiovascular morbidity and mortality.

## Figures and Tables

**Table 1:** Anthropometric and body composition characterization pre- and post-intervention.

	Group 1a (*n* = 27)					Group 1b (*n* = 32)					Group 2 (*n* = 29)					*p* *
	Pre-Intervention		Post-Intervention		*p*	Pre-Intervention		Post-Intervention		*p*	Pre-Intervention		Post-Intervention		*p*	
	Mean (SD)	Median (IQR)	Mean (SD)	Median (IQR)		Mean (SD)	Median (IQR)	Mean (SD)	Median (IQR)		Mean (SD)	Median (IQR)	Mean (SD)	Median (IQR)		
Weight (kg)	71.82 (16.46)	68.20 (17.70)	70.95 (66.15)	66.15 (16.6)	**0.003 ^4^**	72.70 (12.66)	71.33 (17.85)	71.98 (12.65)	69.28 (20.38)	**0.010 ^4^**	69.31 (12.83)	69.05 (14.33)	67.91 (12.14)	66.90 (11.88)	**<0.001 ^4^**	0.459 ^3^
BMI (kg/m^2^)	28.72 (4.89)	28.76 (5.95)	28.38 (4.80)	28.22 (6.60)	**0.003 ^1^**	29.14 (4.32)	28.90 (6.93)	28.85 (4.33)	28.36 (6.59	**0.0017 ^1^**	28.18 (4.41)	28.22 (5.60)	27.63 (4.30)	27.83 (5.20)	**<0.001 ^1^**	0.277 ^2^
Waist circumference (cm)	0.94 (0.13)	0.93 (0.20)	0.92 (0.12)	0.91 (0.18)	0.235 ^4^	0.96 (0.10)	0.96 (0.12)	0.95 (0.09)	0.95 (0.11)	0.246 ^4^	0.90 (0.20)	0.92 (0.17)	0.89 (0.12)	0.88 (0.16)	**0.002 ^4^**	0.063 ^3^
Arm circumference (cm)	31.65 (3.79)	32.00 (5.58)	31.36 (3.48)	31.40 (4.60)	0.234 ^4^	32.04 (3.37)	32.25 (5.88)	31.48 (3.40)	31.90 (5.25)	**<0.001 ^4^**	30.93 (3.09)	30.50 (4.25)	30.61 (3.38)	30.00 (4.80)	**0.028 ^4^**	0.090 ^3^
Bicipital skinfold (mm)	8.89 (5.52)	7.50 (8.50)	8.47 (5.72)	6.25 (8.30)	0.092 ^4^	9.19 (4.55)	9.25 (7.00)	8.76 (4.39)	7.63 (6.94)	0.079 ^4^	10.08 (6.64)	9.10 (5.38)	9.04 (6.93)	7.00 (5.13)	**<0.001 ^4^**	0.085 ^3^
Tricipital skinfold (mm)	18.34 (7.99)	20.00 (11.50)	16.98 (7.64)	17.00 (11.19)	**0.003 ^1^**	18.35 (8.04)	17.18 (13.69)	16.98 (7.04)	16.62 (11.56)	**<0.001 ^1^**	21.11 (8.38)	20.50 (10.38)	18.13 (7.43)	17.25 (7.38)	**<0.001 ^1^**	**0.004 ^2^**
Fat mass (%)	41.27 (8.53)	42.24 (12.20)	39.76 (8.55)	41.48 (11.28)	**<0.001 ^4^**	40.61 (6.92)	41.43 (10.87)	39.38 (6.88)	39.98 (11.03)	**0.002 ^4^**	41.14 (8.04)	41.69 (9.39)	39.23 (7.87)	40.46 (13.53)	**0.002 ^4^**	0.569 ^3^
Muscle mass (%)	25.34 (5.21)	24.04 (5.82)	25.73 (5.44)	24.41 (7.06)	**0.028 ^4^**	26.02 (3.89)	25.26 (5.98)	26.53 (3.86)	26.38 (4.74)	**<0.001 ^4^**	25.05 (4.52)	24.02 (5.14)	25.80 (4.51)	25.13 (4.51)	**0.010 ^4^**	0.422 ^3^
Visceral tissue	2.45 (1.24)	2.20 (1.90)	2.97 (4.01)	2.10 (2.00)	**0.044 ^4^**	3.30 (3.69)	2.40 (1.55)	2.54 (1.35)	2.25 (1.45)	0.085 ^4^	2.40 (1.42)	2.10 (1.40)	1.91 (1.08)	1.50 (1.45)	**<0.001 ^4^**	0.068 ^3^

^1^ Student *t* test for paired samples; ^2^ one-way ANOVA; ^3^ Kruskal–Wallis test; ^4^ Wilcoxon test; BMI: body mass index; SD: standard deviation; IQR: interquartile range. * The rate of change was considered (see statistical analysis in methodology).

**Table 2:** Participants’ analytical values pre- and post-intervention.

	Group 1a (*n* = 27)					Group 1b (*n* = 32)					Group 2 (*n* = 29)					*p* *
	Pre-Intervention		Post-Intervention		*p*	Pre-Intervention		Post-Intervention		*p*	Pre-Intervention		Post-Intervention		*p*	
	Mean (SD)	Median (IQR)	Mean (SD)	Median (IQR)		Mean (SD)	Median (IQR)	Mean (SD)	Median (IQR)		Mean (SD)	Median (IQR)	Mean (SD)	Median (IQR)		
Cholesterol (mg/dL)	189.67 (43.22)	183.00 (55.00)	191.48 (40.31)	191.00 (40.00)	0.733 ^1^	187.06 (40.90)	189.00 (52.25)	175.53 (33.84)	181.00 (52.25)	**0.029 ^1^**	201.55 (46.73)	199.00 (52.50)	195.14 (40.00)	190.00 (46.00)	0.356 ^1^	0.312 ^2^
LDL-C (mg/dL)	104.54 (42.86)	107.00 (58.25)	109.96 (36.20)	108.50 (62.00)	0.115 ^4^	103.13 (32.73)	104.00 (41.00)	96.19 (30.98)	94.00 (51.50)	0.162 ^4^	116.69 (38.35)	112.00 (64.00)	108.17 (28.97)	111.00 (43.00)	0.061 ^4^	**0.010 ^3^**
HDL-C (mg/dL)	56.81 (12.00)	60.00 (16.00)	56.41 (11.57)	56.00 (16.00)	0.672 ^1^	59.66 (17.26)	58.00 (18.75)	56.34 (14.85)	55.00 (18.00)	**0.007 ^1^**	60.24 (13.84)	56.00 (19.00)	61.62 (13.21)	58.00 (18.00)	0.223 ^1^	0.013 ^2^
Triglycerides (mg/dL)	141.63 (88.34)	128.00 (129.00)	128.00 (50.38)	120.00 (66.00)	0.838 ^4^	124.97 (42.94)	122.50 (53.00)	132.56 (58.74)	119.00 (90.75)	0.953 ^4^	132.10 (53.70)	121.00 (72.00)	111.24 (39.58)	104.00 (37.00)	**0.029 ^4^**	0.230 ^3^
Glycated hemoglobin (%)	5.95 (0.96)	5.70 (129.00)	5.84 (0.85)	5.60 (66.00)	0.198 ^4^	5.78 (0.71)	5.60 (0.70)	5.79 (0.56)	5.70 (0.60)	0.605 ^4^	5.73 (0.78)	5.40 (0.75)	5.82 (0.72)	5.60 (0.95)	0.073 ^4^	**0.047 ^3^**
C-reactive protein (mg/L)	9.86 (16.17)	4.25 (2.40)	7.80 (6.83)	3.60 (10.70)	0.593 ^4^	14.07 (15.48)	9.60 (16.50)	6.10 (2.68)	5.55 (4.22)	0.068 ^4^	8.53 (3.76)	8.50 (5.80)	5.74 (3.36)	4.20 (5.40)	0.075 ^4^	0.926 ^3^

^1^ Student *t* test for paired samples; ^2^ one-way ANOVA; ^3^ Kruskal–Wallis test; ^4^ Wilcoxon test; LDL-c: low-density lipoprotein; HDL-c: high-density lipoprotein; CRP: C-reactive protein; SD: standard deviation; IQR: interquartile range. * The rate of change was considered (see statistical analysis in methodology).

**Table 3:** Evaluation of legume/pulse consumption and adherence to the MD at baseline and at the end of the study (post-intervention).

	Group 1a (*n* = 27)					Group 1b (*n* = 32)					Group 2 (*n* = 29)					*p* *
	Pre-Intervention		Post-Intervention		*p*	Pre-Intervention		Post-Intervention		*p*	Pre-Intervention		Post-Intervention		*p*	
	Mean (SD)	Median (IQR)	Mean (SD)	Median (IQR)		Mean (SD)	Median (IQR)	Mean (SD)	Median (IQR)		Mean (SD)	Median (IQR)	Mean (SD)	Median (IQR)		
Nº legumes per week	0.7 (1.2)	0.0 (1.0)	0.6 (1.2)	0.0 (1.0)	0.157 ^2^	1.2 (1.1)	1.0 (2.0)	1.1 (1.2)	1.0 (2.0)	0.257 ^2^	1.0 (1.2)	1.0 (2.0)	4.4 (2.1)	4.0 (4.0)	**<0.001 ^2^**	**<0.001 ^1^**
PREDIMED	8.6 (2.0)	9.0 (2.0)	9.4 (2.2)	10.0 (3.0)	0.101 ^2^	9.1 (1.9)	9.0 (2.0)	8.9 (1.7)	9.0 (3.0)	0.469 ^2^	9.1 (2.1)	9.0 (3.5)	10.1 (2.1)	10.0 (4.0)	**0.024 ^2^**	0.061 ^1^

^1^ Kruskal–Wallis test; ^2^ Wilcoxon test; SD: standard deviation; IQR: interquartile range. * The rate of change was considered (see statistical analysis in methodology).

**Table 4:** Dietary assessment of Group 2 before and after the intervention.

	FREQUENCY				
	Pre-Intervention		Post-Intervention		*p*
	YES	NO	YES	NO	
Do you practice a 12-h fast? *n* (%)	12 (41.4)	17 (58.6)	21 (72.4)	8 (27.6)	**0.007 ^1^**
Do you consume legumes every day? *n* (%)	1 (3.4	28 (96.6)	12 (41.4)	17 (58.6)	**<0.001 ^1^**
Do you consume at most one low-fat dairy product per day? *n* (%)	8 (27.6)	21 (72.4)	22 (75.9)	7 (24.1)	**<0.001 ^1^**
Do you consume 30 grams of nuts per day? *n* (%)	2 (6.9)	27 (93.1)	20 (69.0)	9 (31.0)	**<0.001 ^1^**
Do you consume whole grains? *n* (%)	2 (6.9)	27 (93.1)	12 (41.4)	17 (58.6)	**0.002 ^1^**
Do you consume vegetables in both main meals? *n* (%)	17 (58.6)	12 (41.4)	25 (86.2)	4 (13.8)	**0.005 ^1^**
Do you exclude red meat from your diet? *n* (%)	0 (00.0)	29 (100.0)	11 (37.9)	18 (62.1)	**<0.001 ^1^**
Do you consume alcoholic beverages in moderation? *n* (%)	28 (96.6)	1 (3.4)	29 (100.0)	0 (00.0)	0.317 ^1^
Do you consume potatoes at most twice a week? *n* (%)	10 (34.5)	19 (65.5)	27 (93.1)	2 (6.9)	**<0.001 ^1^**
Do you have at most four meals per day? *n* (%)	20 (69.0)	9 (31.0)	26 (89.7)	3 (10.3)	**0.034 ^1^**

^1^ Wilcoxon test.

## Data Availability

The original contributions presented in the study are included in the article/Supplementary Materials, further inquiries can be directed to the corresponding author/s.

## Supplementary Materials

The following supporting information can be downloaded at: https://www.mdpi.com/article/10.3390/nu16152443/s1, Table S1 Initial characterization of the sample with dropouts. Table S2 Socioeconomic characteristics of study participants. Table S3 Clinical and lifestyle characteristics of study participants. Table S4 Anthropometric characteristics of study participants at baseline. Table S5 Serum biochemical evaluation of participants at the beginning of the study. Table S6 Adherence to the Mediterranean Diet at the beginning of the study. Table S7 Association between variables. Table S8 Presentation of significant results for participants who achieved all dietary goals.
