# Supplementary material for: Impact of a Mediterranean-Inspired Diet on Cardiovascular Disease Risk Factors: A Randomized Clinical Trial
Source: Nutrients. 2024 Jul 26;16(15):2443. doi: 10.3390/nu16152443 (PMC11314620; doi:10.3390/nu16152443)
Supplement: Supplementary file 1 [file nutrients-16-02443-s001.zip › nutrients-3094133-supplementary.pdf]

SUPPLEMENTARY MATERIALS

Table S1 - Initial characterization of the sample with dropouts

Characteristic		All participants (n = 102)
**Age (years), mean ± sd; Median [Q1;Q3]**		69.8 ± 8.3; 72.0 [65.0; 75.3]
**Gender, n (%)**		
	**Female**	74 (72.5)
	**Male**	28 (27.5)
**Cohabitation, n (%)**		
	**Partner**	62 (60.8)
	**Alone**	24 (23.5)
	**Family**	2 (2.0)
	**Other**	14 (13.7)
**Marital status, n (%)**		
	**Married**	66 (64.7)
	**Common-law relationship**	1 (1.0)
	**Single**	4 (3.9)
	**Divorced**	15 (14.7)
	**Widow**	16 (15.7)
**Monthly household income, n %)**		
	**≤500€**	9 (8.8)
	**501 - 1000€**	38 (37.3)
	**1001 – 1500€**	18 (17.6)
	**1501-2000€**	21 (20.6)
	**≥2001€**	16 (15.7)
**Education, n (%)**		
	**No educational level**	1 (1.0)
	**Incomplete 1st cycle**	3 (2.9)
	**1st cycle/4th grade**	37 (36.3)
	**2nd cycle/3rd year of high school**	17 (16.7)
	**3rd cycle/5th year of high school**	13 (12.7)
	**Secondary education**	16 (15.7)
	**Technical-professional course**	3 (2.9)
	**Higher education**	12 (11.8)
**Work situation, n (%)**		
	**Working**	26 (25,5)
	**Retired**	68 (66.7)
	**Unemployed**	6 (5.9)
	**Never worked**	2 (1.9)
**Smoker status, n (%)**		
	**Never smoked**	74 (72.5)
	**Ex-smoker**	21 (20.6)
	**Active smoker**	7 (6.9)
**Alcoholic beverages, n (%)**		36 (35.3)
**Sedentary lifestyle, n (%)**		39 (38.2)
**Reported medications for hypercholesterolemia, n (%)**		47 (46.1)
**Reported medications for hypertension, n (%)**		65 (63.7)
**Family History, n (%)**		32 (31.4)
**Cardiac Insufficiency, n (%)**		5 (4.9)
**Arrhythmia, n (%)**		17 (16.7)
**Stroke, n (%)**		4 (3.9)

**Dyslipidemia, n (%)**	70 (68.6)
**Hypertension, n (%)**	73 (71.6)
**Blood Pressure (mmHg)**	
**Systolic, mean ± sd**	142.9 ± 19.9
**142.0 [130.0; 152.0]**	
**Diastolic, mean ± sd**	79.6 ± 11.9
**79.0 [73.0; 88.0]**	
**BMI, (km/m2), mean ± sd; Median [Q1;Q3]**	28.6 ± 4.3<br>28.5 [25.5; 31.2]
**Normal, n (%)**	20 (19.6)
**Overweight, n (%)**	49 (48.0)
**Obesity, n (%)**	33 (32.4)
**Body weight, (kg), mean ± sd; Median [Q1;Q3]**	71.3 ± 13.7<br>69.7 [61.9; 76.9]
**Fat Mass, (%), mean ± sd; Median [Q1;Q3]**	40.9 ± 7.6<br>41.6 [35.5; 45.9]
**Muscle Mass, (%), mean ± sd; Median [Q1;Q3]**	25.5 ± 4.7<br>24.2 [22.6; 28.3]
**Visceral tissue, mean ± sd; Median [Q1;Q3]**	2.7 ± 2.3<br>2.2 [1.7; 3.2]
**Arm circumference, (cm), mean ± sd; Median [Q1;Q3]**	31.5 ± 3.3<br>31.3 [28.5; 34.2]
**Waist circumference, (cm), mean ± sd; Median [Q1;Q3]**	0.9 ± 0.1<br>0.9 [0.9; 1.0]
**♀ >88 cm, n (%)**	47 (46.1)
**♀ <88 cm, n (%)**	27 (26.5)
**♂ >102 cm, n (%)**	11 (10.8)
**♂ <102 cm, n (%)**	17 (16.6)
**Bicipital skinfold, (mm), mean ± sd; Median [Q1;Q3]**	9.2 ± 5.3<br>8.4 [5.0; 11.6]
**Tricipital skinfold, (mm), mean ± sd; Median [Q1;Q3]**	18.9 ± 8.0<br>19.8 [13.1; 24.1]
**Total cholesterol (mg/dL), mean ± sd; Median [Q1;Q3]**	193.6 ± 42.7<br>191.5 [169.8; 219.0]
**<190, n (%)**	49 (48.0)
**≥190, n (%)**	53 (52.0)
**LDL-C (mg/dL), mean ± sd; Median [Q1;Q3]**	108.9 ± 37.7<br>108.0 [82.0; 132.8]
**<115, n (%)**	56 (54.9)
**≥115, n (%)**	44 (43.1)
**Missing, n (%)**	2 (2.0)
**HDL-C (mg/dL), mean ± sd; Median [Q1;Q3]**	59.2 ± 14.1<br>58.0 [50.8; 68.0]
**♀ >45 mg/dL, n (%)**	71 (69.6)
**♀ <45 mg/dL, n (%)**	3 (2.9)
**♂ >40 mg/dL, n (%)**	21 (20.6)
**♂ <40 mg/dL, n (%)**	7 (6.9)
**Triglycerides (mg/dL), mean ± sd**	131.6 ± 60.6<br>122.5 [91.8; 147.3]
**<150, n (%)**	78 (76.5)
**≥150, n (%)**	24 (23.5)
**Glycated hemoglobin (%), mean ± sd; Median [Q1;Q3]**	5.8 ± 0.8<br>5.6 [5.3; 6.0]
**≤5,6, n (%)**	50 (49.0)
**5,7 – 6,4, n (%)**	28 (27.5)
**≥6,5, n (%)**	14 (13.7)
**Missing, n (%)**	10 (9.8)
**CRP (mg/L), mean ± sd; Median [Q1;Q3]**	10.7 ± 13.0<br>5.4 [3.8; 10.6]
**≤3,0, n (%)**	77 (75.5)
**>3,0, n (%)**	25 (24,5)
**PREDIMED, mean ± sd; Median [Q1;Q3]**	9.1 ± 1.9<br>9.0 [8.0; 10.0]
**Low adherence (≤5), n (%)**	4 (3.9)

**Moderate adherence (6-9), n (%)**	56 (54.9)
**High adherence (≥10), n (%)**	42 (41.2)

Table S2 - Socioeconomic characteristics of study participants

Characteristic	All participants (n = 88)	Group 1a (n = 27)	Group 1b (n = 32)	Group 2 (n = 29)	*p*
**Age (years), mean ± sd**	70.1 ± 7.9<br>72.0 [65.0; 75.0]	69.6 ± 8.3<br>72.0 [62.0; 77.0]	70.9 ± 7.9<br>72.5 [66.0; 75.0]	69.6 ± 7.6<br>72.0 [63.5; 74.5]	0.772[3]
**Gender, n (%)**					
**Female**	65 (73.9)	20 (74.1)	21 (65.6)	24 (82.8)	0.314[1]
**Male**	23 (26.1)	7 (25.9)	11 (34.4)	5 (17.2)	
**Cohabitation, n (%)**					
**Partner**	55 (62.5)	19 (70.4)	19 (59.4)	17 (58.6)	-
**Alone**	21 (23.9)	3 (11.1)	10 (31.3)	8 (27.6)	
**Family**	2 (2.3)	1 (3.7)	0 (0.0)	1 (3.4)	
**Other**	10 (11.4)	4 (14.8)	3 (9.4)	3 (10.3)	
**Marital status, n (%)**					
**Married**	56 (63.6)	20 (74.1)	20 (62.5)	16 (55.2)	-
**Common-law relationship**	1 (1.1)	0 (0.0)	0 (0.0)	1 (3.4)	
**Single**	4 (4.5)	1 (3.7)	1 (3.7)	2 (6.9)	
**Divorced**	14 (15.9)	5 (18.5)	4 (12.5)	5 (17.2)	
**Widow**	13 (14.8)	1 (3.7)	7 (21.9)	5 (17.2)	
**Monthly household income, n %)**					
**≤500€**	8 (9.1)	2 (7.4)	3 (9.4)	3 (10.7)	-
**501 - 1000€**	32 (36.4)	7 (25.9)	8 (25.0)	16 (57.1)	
**1001 – 1500€**	16 (18.2)	5 (18.5)	8 (25.0)	3 (10.7)	
**1501-2000€**	20 (22.7)	8 (29.6)	9 (28.1)	3 (10.7)	
**≥2001€**	12 (13.6)	5 (18.5)	4 (12.5)	3 (10.7)	
**Education, n (%)**					
**No educational level**	1 (1.1)	0 (0.0)	0 (0.0)	1 (3.4)	-
**Incomplete 1st cycle**	3 (3.4)	1 (3.7)	2 (6.3)	0 (0.0)	
**1st cycle/4th grade**	28 (31.8)	6 (22.2)	12 (37.5)	10 (34.5)	
**2nd cycle/3rd year of high school**	17 (19.3)	8 (29.6)	4 (12.5)	5 (17.2)	
**3rd cycle/5th year of high school**	12 (13.6)	4 (14.8)	4 (12.5)	4 (13.8)	
**Secondary education**	14 (15.9)	4 (14.8)	8 (25.0)	2 (6.9)	
**Technical-professional course**	3 (3.4)	0 (0.0)	1 (3.1)	2 (6.9)	
**Higher education**	10 (11.4)	4 (14.8)	1 (3.1)	5 (17.2)	
**Work situation, n (%)**					
**Working**	24 (27.3)	10 (37,0)	9 (28.1)	5 (17.2)	-
**Retired**	56 (63.6)	14 (51.9)	20 (62.5)	22 (75.9)	
**Unemployed**	6 (6.8)	2 (7.4)	2 (6.3)	2 (6.9)	
**Never worked**	2 (2.3)	1 (3.7)	1 (3.1)	0 (0.0)	

Table S3 - Clinical and lifestyle characteristics of study participants

Characteristic	All participants (n = 88)	Group 1a (n = 27)	Group 1b (n = 32)	Group 2 (n = 29)	*P*
**Smoker status, n (%)**					
**Never smoked**	64 (72.7)	20 (74.1)	24 (75.0)	20 (69.0)	
**Ex-smoker**	19 (21.6)	4 (14.8)	8 (25.0)	7 (24.1)	-
**Active smoker**	5 (5.7)	3 (11.1)	0 (0.0)	2 (6.9)	
**Alcoholic beverages, n (%)**	31 (35.2)	8 (29.6)	16 (50.0)	7 (24.1)	0.082[1]
**Sedentary lifestyle, n (%)**	35 (39.8)	15 (55.6)	14 (43.8)	6 (20.7)	**0.024**[1]
**Reported medications for hypercholesterolemia, n (%)**	45 (51.1)	14 (51.9)	15 (46.9)	16 (55.2)	0.808[1]
**Reported medications for hypertension, n (%)**	56 (63.6)	12 (44.4)	26 (81.3)	18 (62.1)	**0.013**[1]
**Family History, n (%)**	29 (33.0)	9 (33.3)	11 (34.4)	9 (31.0)	0.961[1]
**Cardiac Insufficiency, n (%)**	5 (5.7)	1 (3.7)	2 (6.3)	2 (6.9)	0.862[1]
**Arrhythmia, n (%)**	16 (18.2)	3 (11.1)	6 (18.8)	7 (24.1)	0.448[1]
**Stroke, n (%)**	1 (1.1)	0 (0.0)	1 (3.1)	0 (0.0)	0.413[1]
**Dyslipidemia, n (%)**	61 (69.3)	19 (70.4)	19 (59.4)	23 (79.3)	0.239[1]
**Hypertension, n (%)**	60 (68.2)	15 (55.6)	26 (81.3)	19 (65.5)	0.100[1]
**Blood Pressure (mmHg)**					
**Systolic, mean ± sd; Median [Q1;Q3]**	141.6 ± 17.2<br>142.0 [130.0; 152.0]	141.3 ± 16.0<br>141.0 [130.0; 154.0]	142.5 ± 19.9<br>143.0 [127.0; 157.5]	140.8 ± 15.6<br>142.0 [130.5; 150.5]	0.925[2]
**Diastolic, mean ± sd; Median [Q1;Q3]**	78.6 ± 10.6<br>78.0 [73.0; 86.0]	77.0 ± 10.4<br>79.0 [73.0; 82.0]	80.4 ± 10.1<br>80.0 [74.3; 88.8]	77.9 ± 11.5<br>77.0 [72.5; 85.0]	0.544[3]

[1] Chi-Square test; [2] One-Way ANOVA; [3] Kruskal-Wallis test;

Table S4 - Anthropometric characteristics of study participants at baseline

Characteristic	All participants (n = 88)	Group 1a (n = 27)	Group 1b (n = 32)	Group 2 (n = 29)	*p*
**BMI, (km/m2), mean ± sd; Median [Q1;Q3]**	28.7 ± 4.5<br>28.6 [25.4; 31.2]	28.7 ± 4.9<br>28.8 [25.0; 31.0]	29.1 ± 4.3<br>28.9 [25.7; 32.6]	28.2 ± 4.4<br>28.2 [25.1; 30.7]	0.711[2]
**Normal, n (%)**	19 (21.6)	6 (22.2)	6 (18.8)	7 (24.1)	
**Overweight, n (%)**	41 (46.6)	12 (44.4)	15 (46.9)	14 (48.3)	0.973[1]
**Obesity, n (%)**	28 (31.8)	9 (33.3)	11 (34.4)	8 (27.6)	
**Body weight, (kg), mean ± sd; Median [Q1;Q3]**	71.3 ± 13.9<br>69.7 [61.2; 77.2]	71.8 ± 16.5<br>68.2 [60.5; 78.2]	72.7 ± 12.7<br>71.3 [62.5; 80.4]	69.3 ± 12.8<br>69.1 [61.8; 76.1]	0.599[3]
**Fat Mass, (%), mean ± sd; Median [Q1;Q3]**	41.0 ± 7.7<br>41.6 [36.1; 45.9]	41.3 ± 8.5<br>42.2 [35.6; 47.8]	40.6 ± 6.9<br>41.4 [34.9; 45.7]	41.1 ± 8.0<br>41.7 [36.9; 46.3]	0.849[3]
**Muscle Mass, (%), mean ± sd; Median [Q1;Q3]**	25.5 ± 4.5<br>25.5 [24.2; 28.0]	25.3 ± 5.2<br>24.0 [22.0; 27.8]	26.0 ± 3.9<br>25.3 [23.5; 29.5]	25.0 ± 4.5<br>24.0 [22.6; 27.7]	0.294[3]
**Visceral tissue, mean ± sd; Median [Q1;Q3]**	2.7 ± 2.5<br>2.2 [1.7; 3.2]	2.5 ± 1.2<br>2.2 [1.4; 3.3]	3.3 ± 3.7<br>2.4 [1.9; 3.5]	2.4 ± 1.4<br>2.1 [1.5; 2.9]	0.320[3]
**Arm circumference, (cm), mean ± sd; Median [Q1;Q3]**	31.6 ± 3.4<br>31.3 [28.5; 34.4]	31.6 ± 3.8<br>32.0 [28.5; 34.0]	32.0 ± 3.4<br>32.3 [29.1; 35.0]	30.9 ± 3.1<br>30.5 [28.3; 32.5]	0.330[3]
**Waist circumference, (cm), mean ± sd; Median [Q1;Q3]**	0.9 ± 0.1<br>0.9 [0.9; 1.0]	0.94 ± 0.1<br>0.9 [0.8; 1.1]	0.96 ± 0.1<br>1.0 [0.9; 1.0]	0.90 ± 0.2<br>0.9 [0.8; 1.0]	0.332[3]
**♀ >88 cm, n (%)**	43 (66.2)	12 (44.5)	16 (50.0)	15 (51.7)	
**♀ <88 cm, n (%)**	22 (33.8)	8 (29.6)	5 (15.6)	9 (31.0)	
**♂ >102 cm, n (%)**	8 (34.8)	3 (11.1)	3 (9.4)	2 (6.9)	0.953[1]
**♂ <102 cm, n (%)**	15 (65.2)	4 (14.8)	8 (25.0)	3 (10.4)	
**Bicipital skinfold, (mm), mean ± sd; Median [Q1;Q3]**	9.4 ± 5.6<br>8.7 [5.0; 11.9]	8.9 ± 5.5<br>7.5 [4.1; 12.6]	9.2 ± 4.6<br>9.3 [5.3; 12.3]	10.1 ± 6.6<br>9.1 [5.4; 10.8]	0.754[3]
**Tricipital skinfold, (mm), mean ± sd; Median [Q1;Q3]**	19.3 ± 8.2<br>20.0 [13.2; 25.6]	18.3 ± 8.0<br>20.0 [11.8; 23.3]	18.4 ± 8.0<br>17.2 [12.4; 26.1]	21.1 ± 8.4<br>20.5 [16.1; 26.5]	0.336[2]

[1] Chi-Square test; [2] One-Way ANOVA; [3] Kruskal-Wallis test; ♀ female; ♂ male; sd: standard deviation

Table S5 - Serum biochemical evaluation of participants at the beginning of the study

Characteristic	All participants (n = 88)	Group 1a (n = 27)	Group 1b (n = 32)	Group 2 (n = 29)	*p*
**Total cholesterol (mg/dL), mean ± sd; Median [Q1;Q3]**	192.6 ± 43.6<br>190.5 [168.3; 219.0]	189.7 ± 43.2<br>183.0 [167.0; 222.0]	187.1 ± 40.9<br>189.0 [160.3; 212.5]	201.6 ± 46.7<br>199.0 [173.0; 225.5]	0.398[2]
**<190, n (%)**	43 (48.9)	17 (63.0)	16 (50.0)	10 (34.5)	0.102[1]
**≥190, n (%)**	45 (51.1)	10 (37.0)	16 (50.0)	19 (65.5)	
**LDL-C (mg/dL), mean ± sd; Median [Q1;Q3]**	108.1 ± 38.0<br>107.0 [82.0; 132.0]	104.5 ± 42.9<br>107.0 [73.3; 131.5]	103.1 ± 32.7<br>104.0 [82.0; 123.0]	116.7 ± 38.4<br>112.0 [87.5; 150.5]	0.460[3]
**<115, n (%)**	50 (56.8)	16 (59.3)	19 (59.4)	15 (51.7)	0.796[1]
**≥115, n (%)**	38 (43.2)	11 (40.7)	13 (40.6)	14 (48.3)	
**HDL-C (mg/dL), mean ± sd; Median [Q1;Q3]**	59.0 ± 14.6<br>58.0 [50.0; 65.0]	56.8 ± 12.0<br>60.0 [47.0; 63.0]	59.7 ± 17.3<br>58.0 [48.5; 67.3]	60.2 ± 13.8<br>56.0 [51.5; 70.5]	0.649[2]
**♀ >45 mg/dL, n (%)**	62 (95.4)	19 (70.4)	20 (62.5)	23 (79.3)	-
**♀ <45 mg/dL, n (%)**	3 (4.6)	1 (3.7)	1 (3.1)	1 (3.5)	
**♂ >40 mg/dL, n (%)**	17 (73.9)	5 (18.5)	8 (25.0)	4 (13.7)	
**♂ <40 mg/dL, n (%)**	6 (26.1)	2 (7.4)	3 (9.4)	1 (3.5)	
**Triglycerides (mg/dL), mean ± sd; Median [Q1;Q3]**	132.4 ± 63.0<br>122.5 [91.3; 146.8]	141.6 ± 88.3<br>128.0 [75.0; 204.0]	125.0 ± 42.9<br>122.5 [91.8; 144.8]	132.1 ± 53.7<br>121.0 [97.0; 169.0]	0.978[3]
**<150, n (%)**	67 (76.1)	19 (70.4)	26 (81.3)	22 (75.9)	0.620[1]
**≥150, n (%)**	21 (23.9)	8 (29.6)	6 (18.8)	7 (24.1)	
**Glycated hemoglobin (%), mean ± sd; Median [Q1;Q3]**	5,8 ± 0.8<br>5.6 [5.3; 6.1]	5.9 ± 1.0<br>5.7 [5.3; 6.3]	5.8 ± 0.7<br>5.6 [5.3; 6.0]	5.7 ± 0.8<br>5.4 [5.3; 6.1]	0.732[3]
**≤5,6, n (%)**	44 (50.0)	10 (37.0)	17 (53.1)	17 (58.6)	-
**5,7 – 6,4, n (%)**	25 (28.4)	6 (22.2)	10 (31.3)	9 (31.0)	
**≥6,5, n (%)**	12 (13.6)	4 (14.8)	5 (15.6)	3 (10.3)	
**Missing, n (%)**	7 (8.0)	7 (25.9)	0 (0.0)	0 (0.0)	
**CRP (mg/L), mean ± sd; Median [Q1;Q3]**	11.1 ± 13.1<br>5.8 [3.9; 10.6]	9.9 ± 16.2<br>4.3 [3.3; 5.7]	14.1 ± 15.5<br>9.6 [3.8; 20.3]	8.5 ± 3.8<br>8.5 [4.8; 10.6]	0.131[3]
**≤3,0, n (%)**	64 (72.7)	19 (70.4)	23 (71.9)	22 (75.9)	-
**>3,0, n (%)**	24 (27.3)	8 (29.6)	9 (28.1)	7 (24.1)	

[1] Chi-Square test; [2] One-Way ANOVA; [3] Kruskal-Wallis test; ♀ female; ♂ male; LDL-c: low-density lipoprotein; HDL-c: high-density lipoprotein; CRP: C-reactive protein; sd: standard deviation

Table S6 - Adherence to the Mediterranean Diet at the beginning of the study

Characteristic	All participants (n = 88)	Group 1a (n = 27)	Group 1b (n = 32)	Group 2 (n = 29)	*p*
**PREDIMED, mean ± sd; Median [Q1;Q3]**	9.0 ± 2.0<br>9.0 [8.0; 10.0]	8.6 ± 2.0<br>9.0 [8.0; 10.0]	9.1 ± 1.9<br>9.0 [8.0; 10.0]	9.1 ± 2.1<br>9.0 [7.5; 11.0]	.552[2]
**Low adherence (≤5), n (%)**	4 (4.5)	1 (3.7)	2 (6.3)	1 (3.4)	
**Moderate adherence (6-9), n (%)**	51 (58.0)	17 (63.0)	18 (56.3)	16 (55.2)	.947[1]
**High adherence (≥10), n (%)**	33 (37.5)	9 (33.3)	12 (37.5)	12 (41.4)	

[1] Chi-Square test; [2] Kruskal-Wallis test; sd: standard deviation

Table S7 - Association between variables

	Colesterol (mg/dl)	LDL-C (mg/dl)	HDL-C (mg/dl)	Triglycerides (mg/dL)	C-reactive protein (mg/L)	Glycated hemoglobina (%)	PREDIMED SCORE	Atingiu objetivos	Nº legumes per week	12h jejum
**Weight (kg)**	**r_s = - 0.220 ; p = 0.040**	**r_s =-0.333 ; p = 0.002**	**r_s =-0.326 ; p = 0.002**	**r_s = 0.258; p = 0.015**	r_s =-0.159 ; p = 0.503	**r_s = 0.231 ; p = 0.030**	r_s = -0.149 ; p = 0.167	**r_s = - 0.222 ; p = 0.038**	r_s = - 0.124 ; p = 0.250	r_s = 0.012 ; p = 0.913
**BMI (kg/m2)**	r_s = - 0.119 ; p = 0.271	**r_s = - 0.336 ; p = 0.001**	**r_s = - 0.222 ; p = 0.038**	**r_s = 0.355 ; p <.001**	r_s = - 0.290 ; p = 0.215	**r_s = 0.449 ; p <.001**	**r_s = - 0.268 ; p = 0.012**	**r_s = - 0.229 ; p = 0.032**	r_s = - 0.134 ; p = 0.213	r_s = - 0.006 ; p =0.957
**Waist circumference (cm)**	r_s = - 0.205 ; p = 0.056	**r_s = - 0.286 ; p = 0.007**	**r_s = - 0.351 ; p <.001**	**r_s = 0.348 ; p <.001**	r_s = - 0.228 ; p = 0.334	**r_s = 0.368 ; p <.001**	**r_s = - 0.279 ; p = 0.008**	**r_s = - 0.315 ; p = 0.003**	**r_s = - 0.241 ; p = 0.024**	r_s = - 0.125 ; p = 0.247
**Arm circumference (cm)**	r_s = - 0,181 ; p = 0,092	**r_s = - 0,315 ; p = 0,003**	**r_s = - 0,323 ; p = 0,002**	**r_s = 0,227 ; p = 0,034**	r_s = 0,201; p = 0,395	**r_s = 0,272 ; p = 0,010**	**r_s = - 0,288 ; p = 0,006**	**r_s = - 0,302 ; p = 0,004**	r_s = - 0,153 ; p = 0,154	r's = 0,014 ; p = 0,900
**Bicipital skinfold (mm)**	r_s = - 0,125 ; p = 0,249	**r_s = - 0,300 ; p = 0,005**	r_s = - 0.094 ; p = 0.387	**r_s = 0.220 ; p = 0.041**	r_s = 0.092 ; p = 0.698	**r_s = 0.531 ; p < .001**	**r_s = - 0.389 ; p < .001**	r_s = - 0.096 ; p = 0.378	r_s = - 0.067 ; p = 0.536	r_s = 0.086 ; p = 0.426
**Tricipital skinfold (mm)**	r_s = 0.016 ; p = 0.885	r_s = - 0.202 ; p = 0.062	r_s = 0.090 ; p = 0.407	r_s = 0.202 ; p = 0.061	r_s = - 0.0.14 ; p = 0.955	**r_s = 0.443; p < .001**	r_s = - 0.206 ; p = 0.056	r_s = - 0.039 ; p = 0.723	r_s = 0.028 ; p = 0.798	r_s = 0.089 ; p = 0.411
**Fat Mass (%)**	r_s = - 0.080; p = 0.461	**r_s = - 0.283; p = 0.008**	r_s = - 0.145; p = 0.178	**r_s = 0.384; p < .001**	r_s = - 0.221; p = 0.348	**r_s = 0.398; p < .001**	**r_s = - 0.318; p = 0.003**	r_s = - 0.160; p = 0.136	r_s = - 0.106; p = 0.327	r_s = 0.047; p = 0.666
**Muscle Mass (%)**	r_s = - 0.146; p = 0.176	r_s = 0.016; p = 0.885	**r_s = - 0.317; p = 0.003**	r_s = - 0.187; p = 0.081	r_s = 0.275; p = 0.241	**r_s = - 0.215; p = 0.044**	r_s = 0.164; p = 0.127	r_s = - 0.062; p = 0.567	r_s = - 0.067; p = 0.533	r_s = - 0.147; p = 0.171
**Visceral tissue**	r_s = - 0.200; p = 0.062	**r_s = - 0.221; p = 0.040**	**r_s = - 0.352; p < .001**	**r_s = 0.271; p = 0.011**	r_s = - 0.091; p = 0.703	**r_s = 0.263; p = 0.013**	r_s = - 0.185; p = 0.084	**r_s = - 0.282; p = 0.008**	**r_s = - 0.253; p = 0.017**	r_s = - 0.096; p = 0.375
**Muscle Area Arm**	r_s = 0.044; p = 0.683	r_s = - 0.121; p = 0.267	r_s = 0.046; p = 0.670	r_s = 0.206; p = 0.055	r_s = - 0.209; p = 0.377	**r_s = 0.381; p < .001**	r_s = - 0.028; p = 0.800	r_s = 0.060; p = 0.580	r_s = 0.102; p = 0.349	r_s = 0.103; p = 0.341
**PREDIMED SCORE**	r's = 0.139; p = 0.197	**r_s = 0.243; p = 0.023**	r_s = 0.125; p = 0.247	r_s = - 0.142; p = 0.188	r_s = - 0.011 ; p = 0.963	**r_s = - 0.255; p = 0.017**	-	-	-	-
**Atingiu objetivos**	r_s = 0.172; p = 0.109	r_s = 0.198; p = 0.065	r_s = 0.171; p = 0.111	r_s = - 0.165; p = 0.125	r_s = - 0.261; p = 0.267	r_s = - 0.186; p = 0.084	-	-	-	-
**Nº legumes per week**	r_s = 0.124; p = 0.248	r_s = 0.136; p = 0.209	r_s = 0.095; p = 0.381	**r_s = - 0.248; p = 0.020**	r_s = - 0.020; p = 0.933	r_s = - 0.127; p = 0.240	-	-	-	-
**12-hour fast**	r_s = 0.005; p = 0.963	r_s = - 0.013; p = 0.902	r_s = 0.072; p = 0.507	r_s = 0.062; p = 0.568	r_s = - 0.131; p = 0.582	r_s = - 0.010; p = 0.930	-	-	-	-

Table S8 - Presentation of significant results for participants who achieved all dietary goals

Attained the dietary objectives?		Triceps skinfold thickness	Muscle mass
	N (%)	74 (84.1)	76 (86.4)
**No**	Mean, sd	-9.53 (11.6)	+ 1.7 (4.3)
	Mediana	-8.51	+ 1.8
	N (%)	12 (13.6)	12 (13.6)
**Yes**	Mean, sd	-21.4 (13.3)	+ 4.5 (4.0)
	Mediana	-27.0	+ 3.8

SD: standard deviation; IQR: interquartile range

*The rate of change was considered [see statistical analysis at methodology]
